# Message for the new year— Life and Hope in 2023

**DOI:** 10.1002/ame2.12308

**Published:** 2023-02-03

**Authors:** Chuan Qin

**Affiliations:** ^1^ Chinese Association for Laboratory Animal Sciences Beijing China; ^2^ Institute of Laboratory Animal Sciences Chinese Academy of Medical Sciences & Peking Union Medical College Beijing China

According to the traditional Chinese zodiac, 2023 is the Year of the Rabbit, which holds the meaning of life and hope due to the rabbit's vigorous reproductive ability.

Over the past 5 years *AMEM* has flourished, initial hopes have been realized and the journal has gone from strength to strength. Following the gradual rise in submissions and the journal's increasing influence, in 2022 AMEM changed from quarterly to bimonthly, and successively launched seven themed sections – columns – including neurodegenerative disease, cardiovascular disease, and so on. In the same year, it was included in the Scopus database, another milestone in the development of AMEM following inclusion in the DOAJ, PubMed Central, ESCI, and MEDLINE databases. In line with its remit of combining animal models and experimental medicine, *AMEM* publishes original articles, reviews, research highlights, reviews and other different types of articles on laboratory animals and animal experimental research to meet the needs of readers. As well as disseminating excellent achievements in experimental and translational medicine, the journal has always been committed to providing an international platform for reporting more valuable laboratory animal models.

*AMEM* is the first international English‐language journal in China devoted to innovation and development of laboratory animal science and experimental medicine, including reporting on animal welfare and hot issues of concern to the Asian Federation of Laboratory Animal Science Associations. AMEM took the lead in publishing the *Guidelines for Ethical Review of Laboratory Animal Welfare in China in 2020*, demonstrating to the world the normativity and professionalism of animal experiments in China. As a journal with a sense of social responsibility, at a critical time with COVID‐19 raging around the world and endangering human health, the *AMEM* editorial team published the world's first research results from a rhesus monkey COVID‐19 model in the first issue in March 2020 (cited 174 times). *AMEM* has subsequently published a number of research papers on hot and difficult issues related to COVID‐19, including a column on the interaction between viruses and hosts in October 2022, allowing people around the world to see the research results of Chinese scientists.

As well as publishing work from China, AMEM also includes reports from Asia and the rest of the world. As the first English journal in the field of laboratory animal research in China, it shoulders the great responsibility of facilitating exchange, reference and complementarity between China and the world in many fields such as basic research on laboratory animals, breeding, welfare and ethics, transformation and application. We are pleased to see that more and more articles from international peers are being published in *AMEM* and we hope that the proportion of these articles will continue to increase in the future.

The prevention and control of COVID‐19 has entered the post‐epidemic stage and there will be much more scientific work of increasing importance to be completed in the future. In many areas of human diseases and research and development on drugs, there are still difficult problems that need to be solved. Animal models are an important part of laboratory animal science, and are widely used in experimental medicine for simulating human diseases. On the road to optimizing and innovating animal models and finding models closer to human diseases, scientists have never stopped exploring, and we are full of hope that we can meet new challenges. I sincerely hope that with the help of experts in the publishing industry and the editorial department, *AMEM* will move to a broader stage and become a well‐known and internationally renowned academic journal, a platform on which the world's outstanding scientists can display their achievements. I anticipate that *AMEM* will achieve more brilliant results in 2023 and wish everyone a happy new year!

